# Effects of Cage Position and Light Transmission on Home Cage Activity and Circadian Entrainment in Mice

**DOI:** 10.3389/fnins.2021.832535

**Published:** 2022-01-10

**Authors:** Laura C. E. Steel, Selma Tir, Shu K. E. Tam, James N. Bussell, Manuel Spitschan, Russell G. Foster, Stuart N. Peirson

**Affiliations:** ^1^Nuffield Department of Clinical Neurosciences, Sir Jules Thorn Sleep and Circadian Neuroscience Institute (SCNi), University of Oxford, Oxford, United Kingdom; ^2^Department of Biomedical Services, University of Oxford, Oxford, United Kingdom; ^3^Max Planck Institute for Biological Cybernetics, Tübingen, Germany; ^4^TUM Department of Sport and Health Sciences (TUM SG), Technical University of Munich, Munich, Germany

**Keywords:** light, circadian, retina, melanopsin, cage rack, individually ventilated cage (IVC), reproducibility

## Abstract

Light is known to exert powerful effects on behavior and physiology, including upon the amount and distribution of activity across the day/night cycle. Here we use home cage activity monitoring to measure the effect of differences in home cage light spectrum and intensity on key circadian activity parameters in mice. Due to the relative positioning of any individually ventilated cage (IVC) with regard to the animal facility lighting, notable differences in light intensity occur across the IVC rack. Although all mice were found to be entrained, significant differences in the timing of activity onset and differences in activity levels were found between mice housed in standard versus red filtering cages. Furthermore, by calculating the effective irradiance based upon the known mouse photopigments, a significant relationship between light intensity and key circadian parameters are shown. Perhaps unsurprisingly given the important role of the circadian photopigment melanopsin in circadian entrainment, melanopic illuminance is shown to correlate more strongly with key circadian activity parameters than photopic lux. Collectively, our results suggest that differences in light intensity may reflect an uncharacterized source of variation in laboratory rodent research, with potential consequences for reproducibility. Room design and layout vary within and between facilities, and caging design and lighting location relative to cage position can be highly variable. We suggest that cage position should be factored into experimental design, and wherever possible, experimental lighting conditions should be characterized as a way of accounting for this source of variation.

## Introduction

Light exerts many effects on behavior including regulating the amount and distribution of locomotor activity. This includes the acute regulation of activity by light (masking) as well as the synchronization of circadian rhythms with the external light/dark cycle. Circadian rhythms are endogenous near-24 h rhythms in physiology and behavior that persist under constant conditions, providing evidence for an internally generated biological clock. These rhythms provide a selective advantage by enabling anticipation of the rhythmically changing environment across the day/night cycle, and the realignment of physiology to exploit these differences. In mammals, the master circadian clock is located in the suprachiasmatic nuclei (SCN) in the anterior hypothalamus, where cell-autonomous rhythms are generated via an intracellular transcriptional-translational feedback loop, consisting of a number of core clock genes (Hastings et al., 2018). However, circadian clocks are also found in cells and tissues throughout the body, and the SCN is thought to act as a pacemaker to coordinate these peripheral clocks to ensure they maintain an appropriate phase (Dibner et al., 2010).

Circadian rhythms are not exactly 24 h and in nocturnal rodents such as mice they are slightly shorter than 24 h. As such, daily adjustment of the circadian clock is required so that internal circadian time is appropriately phased with external environmental time. The primary time cue (zeitgeber) for this process of entrainment is light, which in all mammals is detected by the eye. Research on the retinal photoreceptors mediating circadian responses to light has led to the discovery of a new photoreceptor system, in addition to classical rod and cone photoreceptors that mediate vision. This consists of a subset of photosensitive retinal ganglion cells (pRGCs) expressing the blue-light sensitive photopigment melanopsin (Foster et al., 2020). These cells project to the SCN as well as numerous other brain regions, regulating many different non-visual responses to light (Hughes et al., 2016).

The effects of light on locomotor activity—both in terms of masking and circadian rhythms—are dose dependent. Studies on the threshold of circadian entrainment have shown that mice are remarkably sensitive to light, and are able to entrain down to light levels around 0.01 photopic lux (Ebihara and Tsuji, 1980; Butler and Silver, 2011); although there is some variation between strains of mice, with C57 mouse strains more sensitive than C3H (Foster and Helfrich-Förster, 2001). Increasing light intensities lead to greater activity suppression in response to light (Mrosovsky, 1999; Thompson et al., 2008; Contreras et al., 2021) and greater circadian phase shifts (Foster et al., 1991; Yoshimura et al., 1994; Provencio and Foster, 1995; Hattar et al., 2003). These responses are typically plotted as irradiance-response curves, with increasing light levels resulting in greater biological responses up to a point of saturation. The effects of the wavelength are to shift the relative position of these irradiance-response curves, requiring more or less light to evoke the same response (corresponding to lower or higher sensitivity, respectively). Such studies have shown that mice are most sensitive to light around 480–511 nm (Provencio and Foster, 1995; Yoshimura and Ebihara, 1996; Hattar et al., 2003; Peirson et al., 2005). Moreover, these studies clearly show that mice can respond to longer wavelength light, but since they lack a long-wavelength sensitive (LWS) cone like humans, they are relatively less sensitive to red light in comparison with humans (Peirson et al., 2018). Together these data illustrate that the use of photopic lux—a measurement of illuminance based upon human visual sensitivity which peaks around 555 nm—are inappropriate for measuring the effects of light on mice. However, to date, most guidance on light levels in animal facilities are given in photopic lux.

Light levels differ markedly across mouse cage racks and reflect a source of potential experimental variability. These differences can be up to 80-fold between cages on the top and bottom of a cage rack in normal transparent cages (Clough, 1982). Individually ventilated cages (IVCs) are widely used in laboratory mouse husbandry, offering many advantages including increased biosecurity, stocking density, controlled environmental conditions, and reduced exposure to laboratory animal allergens (Brandstetter et al., 2005). IVC design varies between distributors, with differences in materials as well as the spacing of cages within a rack. IVC racks house multiple rows of cages, and cages may be produced to reduce in-cage light exposure. As such, the light levels experienced by any animal within an IVC rack will depend upon the cage position as well as the cage material. Both the row and column of a cage may affect the light intensity, depending upon how the rack is positioned relative to the room lighting (Clough et al., 1995).

Although light is known to mediate both acute and circadian effects on the regulation of locomotor activity, to date no studies have systematically evaluated the effects of cage position on light and home cage activity. Here we describe the effects of cage position and filtering on cage light levels, and the effects of these systematic differences of lighting on home cage activity using the Digital Ventilated Cage (DVC) system (Tecniplast). We predicted that mice housed in cages with lower light levels will show less stable circadian entrainment and more daytime activity.

## Materials and Methods

### Animals

This study had an initial cohort of 12 WT (6 female and 6 male) mice of C57BL/6J background (Envigo, Blackthorn, United Kingdom, RRID:IMSR_ JAX:000664), aged 11-weeks at the start of the experiment. However, one male was culled prior to the start of experiments due to fighting injuries. All mice were singly housed in new cages (that had undergone minimal processing to meet the hygiene levels of the facility) and these cages were not changed for the duration of the experiment. All cages were placed in the Digital Ventilated Cage (DVC) rack (Tecniplast, Italy) at 20–24°C and 45–65% ±10% humidity, with food (Envigo 2916) and water available *ad libitum*. Mice were maintained under a cool white fluorescent light source with a ramped 13 h 10-min/10 h 50-min light-dark cycle [lights on at 07:50 and reaching full intensity (260 photopic lux) at 09:00, and fully off at 21:00]. Animals were housed in specific pathogen free (SPF) conditions, and the only prior reported positives on health screening in this unit were *Entamoeba muris, Enteromonas* Sp., and *Trichomonas* Sp.; though no positives were reported during the course of this study (Envigo, Alconbury, United Kingdom). All experimental procedures were conducted at the University of Oxford, United Kingdom in accordance with the United Kingdom Animals (Scientific Procedures) Act 1986 under Project License PP0911346 and Personal License I82616702.

### Experimental Design

The cohort of 11 animals was randomly split into two groups. There was a control group of 5 mice (3 female and 2 male) which were housed in standard Green Line individually ventilated cages (IVC; Tecniplast GM500, polysulfone), and an experimental group of 6 mice (3 female and 3 male) which were housed in red individually ventilated cages (IVC; Tecniplast GM500, polysulfone). All mice were habituated in the central rows of the DVC rack for 1 week prior to the start of the experiment. Animals were then assigned to “top”, “middle”, and “bottom” row positions within the DVC rack (Supplementary Figure 1), spending 1 week at each position. Mice were rotated through the different rack positions in a counterbalanced order and were culled via cervical dislocation at the end of the 3 week experimental period.

### Home Cage Activity Monitoring

All mice were singly housed in a Digital Ventilated Cage (DVC) rack (Tecniplast). This is an IVC rack which continuously measures activity via capacitance sensing technology (CST) (Iannello, 2019). A sensing board is installed below each IVC cage in the rack and is composed of 12 equally spaced electrodes, the electrical capacitance of which are measured every 250 ms. Due to the high-water content of animals, the capacitance of the electrodes is significantly influenced by the presence of mice. Therefore, animal movement can be recorded as changes in capacitance across electrodes (Iannello, 2019; Pernold et al., 2019). Capacitance measurements from adjacent electrodes are compared, and when the difference is larger than a fixed threshold the electrode is considered activated. From this, an animal locomotion index (ALI) is produced which is expressed in an arbitrary unit normalized between 0 and 100%. 0% represents no activity, and if all electrodes are simultaneously activated, a value of 100% is produced. For our analysis, we exported the animal locomotion index from DVC Analytics, in 1 min bins across all 12 electrodes.

### Light Measurements

**FIGURE 1:**
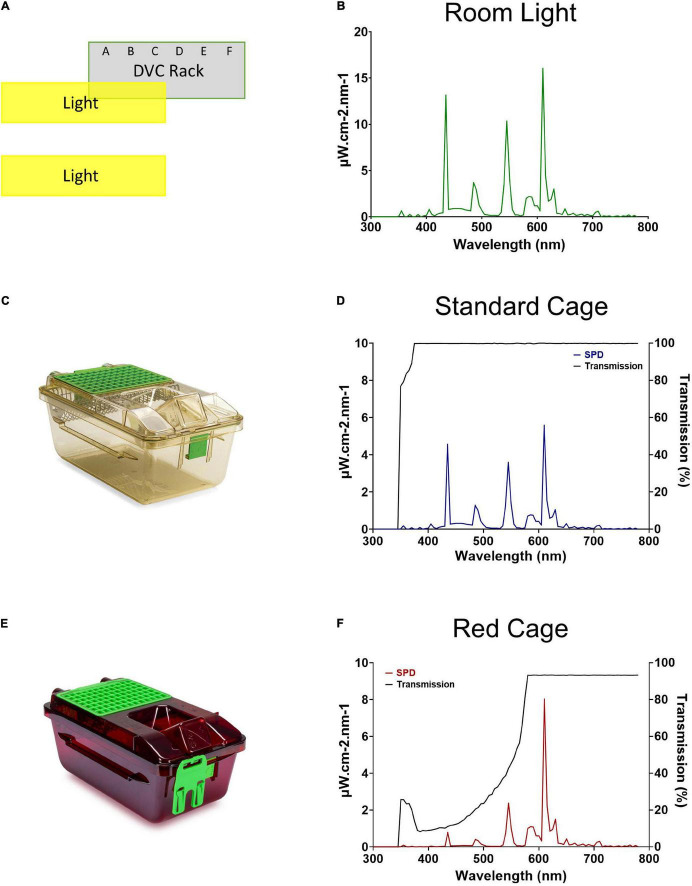
Room lighting and cage transmission. **(A)** Schematic of the relative positioning of the DVC rack and the room lights. **(B)** Spectral power distribution of the DVC room light. **(C)** Standard Green Line individually ventilated cage (IVC; Tecniplast GM500). **(D)** Spectral power distribution of the internal light environment, and the transmission, of a standard cage. **(E)** Red individually ventilated cage (IVC; Tecniplast GM500). **(F)** Spectral power distribution of the internal light environment, and the transmission, of a red cage.

A XL-500 BLE Spectroradiometer (NanoLambda, Korea) was used to take power and photopic lux measurements of the internal light environment of the standard and red cages in positions across the DVC rack. Individual measurements were taken in the front, middle and back of each cage, in all columns across the top (row 10), middle (row 6), and bottom (row 1) of the DVC rack (Supplementary Table 1). The mean of these within cage values were taken, to produce a single measure of photopic lux for each cage type across the relevant positions of the DVC rack (Table 1). The spectral power distribution (SPD) of the room light where the DVC rack was located, was measured at the height of the top row of the DVC rack using a calibrated Ocean Optics USB2000+ Spectrophotometer (Ocean Insight, Oxford, United Kingdom). Standard and red cage transmission measurements were taken under dark conditions, using a calibrated Ocean Optics Spectrometer and a broad-spectrum microscope light source (Photonic, Wein, Austria). The DVC room light spectral power distribution was corrected by these cage transmission measurements to produce relative spectral power distributions (RSPD). Linear interpolation was used to convert these RSPDs into 5 nm bins, and α-opic irradiance values (CIE, 2018) were then calculated using the rodent toolbox for S- and M- cones, rods, and melanopsin for each cage position (Lucas et al., 2014). Due to the positioning of the room lights in relation to the DVC rack, as shown in Figure 1A, the light intensity varied significantly across the DVC rack—showing in general, a decrease in intensity from top left to bottom right of the rack (Supplementary Figure 2).

**TABLE 1:** Light intensity in photopic lux and other α-opic irradiance, in each column (A–F) of the DVC rack across the top, middle, and bottom rows.

Standard cage irradiance	A	B	C	D	E	F
Top—Photopic	177.5	145.5	122.9	46.5	54.1	29.7
Top—S-cone	19.0	15.6	13.2	5.0	5.8	3.2
Top—Melanopic	113.3	92.9	78.5	29.7	34.5	19.0
Top—Rhodopic	115.2	94.4	79.7	30.2	35.1	19.3
Top—M-cone	119.6	98.0	82.8	31.3	36.4	20.0
Middle—Photopic	39.6	70.4	69.6	57.7	44.6	20.3
Middle—S-cone	4.2	7.5	7.5	6.2	4.8	2.2
Middle—Melanopic	25.3	44.9	44.4	36.8	28.5	13.0
Middle—Rhodopic	25.7	45.7	45.2	37.4	28.9	13.2
Middle—M-cone	26.7	47.4	46.9	38.9	30.0	13.7
Bottom—Photopic	17.3	13.4	18.6	13.3	11.6	13.7
Bottom—S-cone	1.9	1.4	2.0	1.4	1.2	1.5
Bottom—Melanopic	11.0	8.6	11.9	8.5	7.4	8.7
Bottom—Rhodopic	11.2	8.7	12.1	8.6	7.5	8.9
Bottom—M-cone	11.7	9.0	12.5	9.0	7.8	9.2

**Red cage irradiance**	**A**	**B**	**C**	**D**	**E**	**F**

Top—Photopic	19.1	16.9	13.5	6.2	6.5	3.3
Top—S-cone	0.7	0.6	0.5	0.2	0.2	0.1
Top—Melanopic	4.4	3.9	3.1	1.4	1.5	0.8
Top—Rhodopic	5.4	4.8	3.8	1.8	1.8	0.9
Top—M-cone	5.9	5.2	4.1	1.9	2.0	1.0
Middle—Photopic	6.0	8.8	7.9	6.9	5.3	2.6
Middle—S-cone	0.2	0.3	0.3	0.2	0.2	0.1
Middle—Melanopic	1.4	2.0	1.8	1.6	1.2	0.6
Middle—Rhodopic	1.7	2.5	2.2	2.0	1.5	0.7
Middle—M-cone	1.8	2.7	2.4	2.1	1.6	0.8
Bottom—Photopic	3.9	3.7	3.4	3.5	2.8	1.8
Bottom—S-cone	0.1	0.1	0.1	0.1	0.1	0.1
Bottom—Melanopic	0.9	0.9	0.8	0.8	0.6	0.4
Bottom—Rhodopic	1.1	1.0	1.0	1.0	0.8	0.5
Bottom—M-cone	1.2	1.1	1.9	1.1	0.9	0.6

*Reported for a standard cage (top table) and red cage (bottom table).*

### Circadian Activity Measures

The animal locomotion index, for all 11 cages across the 3 week period, was exported from DVC Analytics in 1 min bins and processed in Matlab (R2021a). Following this, Matlab (R2021a) and Actogram J (Schmid et al., 2011) were used to calculate several circadian activity measures for each mouse in each position (Brown et al., 2019). These are described below. For all analyses, the light phase was defined as 07:50–20:59 and the dark phase from 21:00 to 07:49. Zeitgeber time (ZT) 0 was defined as light onset (07:50 local clock time).

#### Light and Dark Phase Activity (%)

In the laboratory, WT mice are nocturnal, with activity mainly limited to the dark phase. An increase in the proportion of activity carried out during the light phase is therefore considered a marker of circadian disruption (Oliver et al., 2012). Matlab (R2021a) was used to calculate light and dark phase activity, expressed as a percentage of total activity across the 24 h period (Brown et al., 2019).

#### Relative Amplitude

The relative amplitude of a circadian rhythm is the difference between periods of peak activity and rest across the 24 h cycle (van Someren et al., 1999; Brown et al., 2019). A low relative amplitude value is indicative of a weak and disrupted circadian rhythm, since it shows fewer distinct and consolidated periods of activity and rest. In our analysis, the active period was defined as the dark phase and the rest period defined as the light phase. The relative amplitude of every 24 h period for each mouse was calculated using Matlab (R2021a), and the mean taken of relevant measures in order to output a single relative amplitude measure for each mouse in the top, middle and bottom DVC rack positions.

#### Activity Onset

An animal with a normal circadian rhythm will begin activity around the same time each day. A small phase angle of entrainment and low variability in activity onset between days can therefore be a marker of circadian robustness (Brown et al., 2019). Activity onset for every 24 h period for each mouse was calculated using Actogram J’s inbuilt function, which first smooths the data (using the standard deviation of a smoothing Gaussian distribution). Following this, activities are considered “active” if they exceed the threshold of the median of all activity values (Schmid et al., 2011). The mean and standard deviation of activity onset for each mouse in the top, middle, and bottom DVC rack positions were calculated in Matlab (R2021a).

#### Regularity Disruption Index

The Regularity Disruption Index (RDI) was developed to quantitatively measure irregular activity patterns and is based on sample entropy (Golini et al., 2020). A high RDI is indicative of a more irregular rhythm, whilst a low RDI suggests reliable activity patterns. This measure can be exported directly from the DVC Analytics website, with separate analysis for the light and dark phase.

#### Activity Bouts

Circadian disruption results in increased fragmentation of activity and rest (Brown et al., 2019), and so is often associated with changes in the proportion of activity bouts of different lengths. Light and dark phase activity were analyzed separately using Matlab (2021a) to categorize activity bouts into bins of different lengths (1, 2, 3, 4–5, 6–10, 11–21, 21–40, and > 40 min). Time weighted frequency histograms were produced (based on analysis from Mochizuki et al., 2004) to explore changes in activity bout distribution.

#### Periodogram Power

Periodogram power is a measure of the strength of a rhythm, with higher values reflecting more reliable rhythms and very low values indicating arrhythmicity (Brown et al., 2019). The power of the chi-squared periodogram (Qp) is particularly commonly used in circadian analysis—if the Qp value for a period exceeds that of the expected background value based on the chi-square distribution, the period is considered significant (Sokolove and Bushell, 1978; Brown et al., 2019). The Qp value for each mouse in each position was calculated using Actogram J’s inbuilt chi-squared periodogram function (Schmid et al., 2011).

#### Inter-Daily Stability

Inter-daily stability (IS) is a measure of the day-to-day reproducibility of activity cycles. Activity patterns are highly reproducible in healthy animals, and so a low IS value suggests circadian disruption. IS was calculated in Matlab (2021a) as the variance of the average 24 h activity pattern expressed as a ratio of total variance (Brown et al., 2019).

#### Intra-Daily Variability

Intra-daily variability (IV) is a measure of the number of transitions between activity and rest—with a higher IV value reflecting a more fragmented rhythm (Brown et al., 2019). IV was calculated in Matlab (2021a).

### Statistical Analysis

GraphPad Prism 9 was used to visualize the data and run statistical analysis. Statistical significance of differences in circadian disruption measures across cage type and position were tested with two-way repeated-measures ANOVAs, with a Greenhouse-Geisser correction applied to adjust for lack of sphericity (Figure 3). Similarly, the relationship between frequency of activity bouts of different lengths, with cage type and position, were also tested with two-way repeated-measures ANOVAs (Figure 4). Irradiance measurements were log10 transformed and a simple linear regression was applied to investigate the relationship between light intensity and circadian activity parameters, for each mouse in each DVC rack position (Figure 5).

**FIGURE 2:**
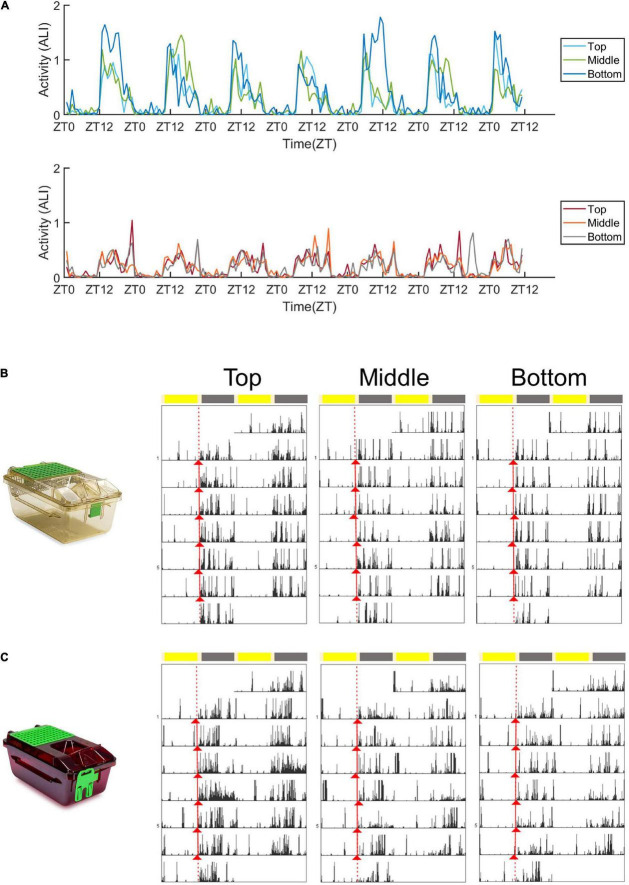
Activity plots and representative actograms. **(A)** Mean activity (measured as the animal locomotion index) of mice housed in standard cages (top panel) and red cages (bottom panel) across 1 week in the top, middle, and bottom positions. **(B)** Double plotted actograms (Actogram J) of a representative standard cage housed mouse in the 1 week spent in the top, middle, and bottom positions of the DVC rack. Activity onsets are marked in red (Actogram J). **(C)** Double plotted actograms (Actogram J) of a representative red cage housed mouse in the 1 week spent in the top, middle, and bottom positions of the DVC rack. Activity onsets are marked in red (Actogram J).

**FIGURE 3:**
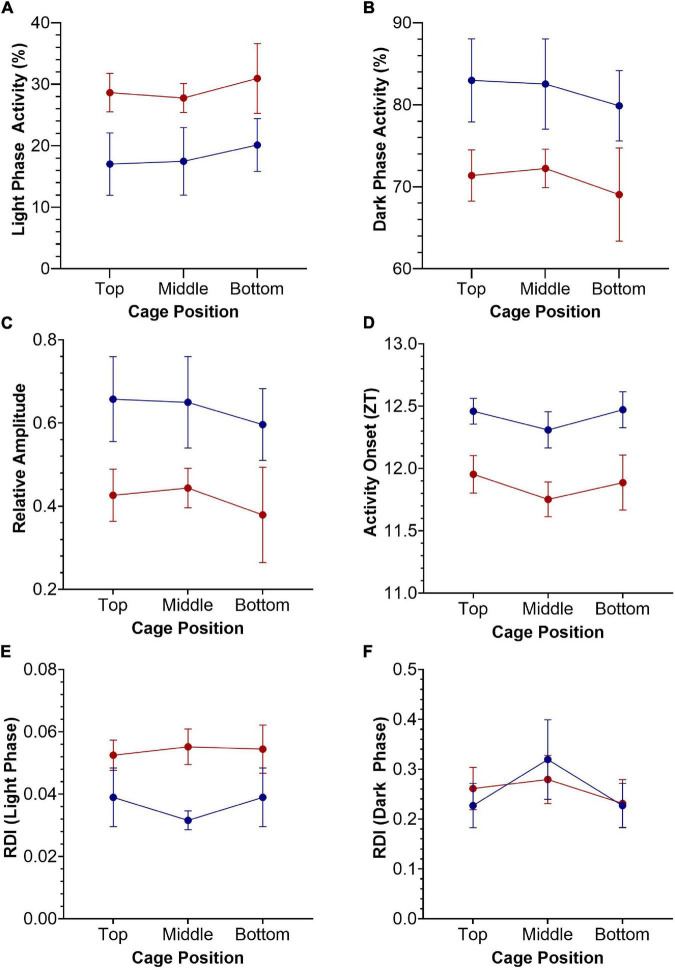
Key circadian activity parameters plotted as mean +/– SEM for standard (blue) and red (red) cages, across top, middle, and bottom positions of the DVC rack. Two-way repeated-measures ANOVAs were used to test for significant effects of cage type and cage position. **(A)** Light phase activity (%), main effect of cage type [*F*_(1.0, 9.0)_ = 4.196, *p* = 0.071]. **(B)** Dark phase activity (%), main effect of cage type [*F*_(1.0, 9.0)_ = 4.196, *p* = 0.071]. **(C)** Relative amplitude, main effect of cage type [*F*_(1.0, 9.0)_ = 4.151, *p* = 0.072]. **(D)** Activity Onset (ZT), significant main effect of cage type [*F*_(1.0, 9.0)_ = 8.264, *p* = 0.018]. **(E)** Regularity disruption index (RDI)—light phase, main effect of cage type [*F*_(1.0, 9.0)_ = 3.883, *p* = 0.080]. **(F)** Regularity disruption index (RDI)—dark phase, significant main effect of cage position [*F*_(1.8, 16.6)_ = 5.545, *p* = 0.016].

**FIGURE 4:**
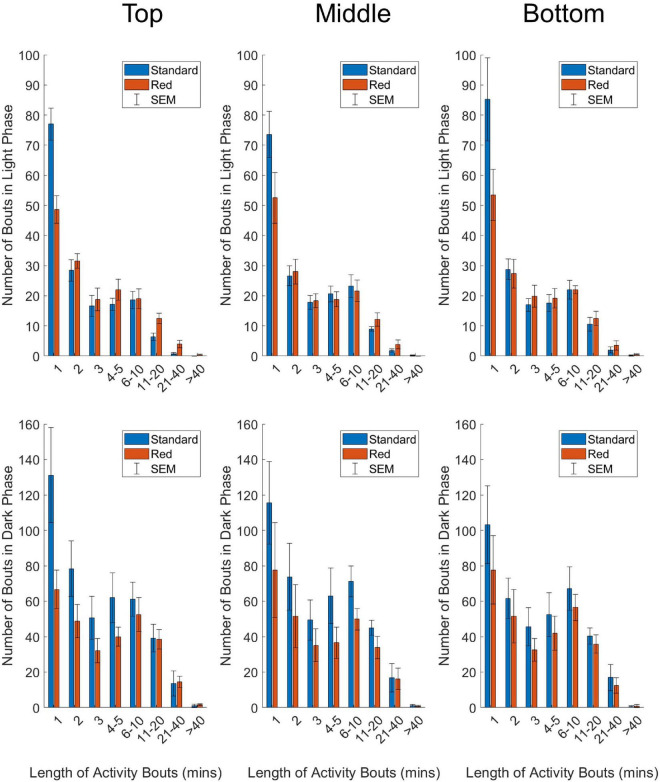
Number of activity bouts of different lengths in standard (blue) and red (red) IVCs, plotted as mean +/– SEM in time-weighted frequency histograms. Analyzed separately for light phase **(top panel)** and dark phase **(bottom panel)** data, and separately by DVC rack position **(top, middle, and bottom)**. Two-way repeated-measures ANOVAs were used to test for significant effects of cage type and bout length on number of bouts. A significant main effect of bout length was reported for all positions, across the light and dark phase. A significant cage type by cage position was reported for all positions in the light phase, and the top position in the dark phase.

**FIGURE 5:**
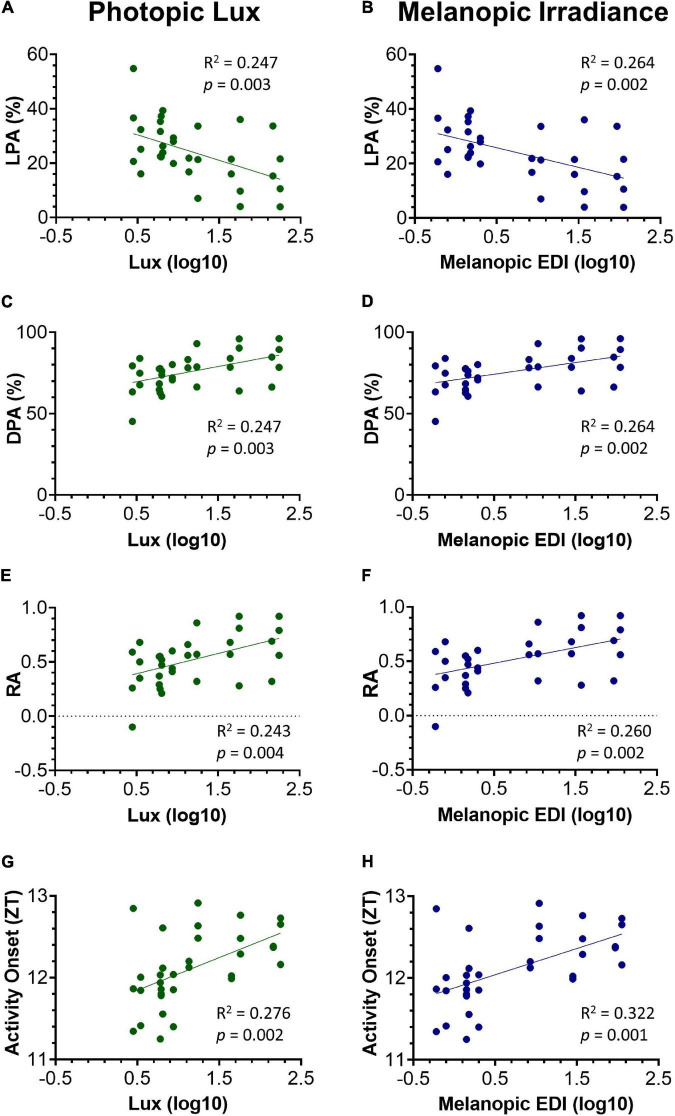
Relationship between key circadian activity parameters and photopic lux **(A,C,E,G)** and melanopic irradiance **(B,D,F,H)**. All light intensity values have been log10 transformed. Light phase activity (LPA) (%) against photopic lux **(A)** and melanopic EDI **(B)**. Dark phase activity (DPA) (%) against photopic lux **(C)** and melanopic EDI **(D)**. Relative amplitude (RA) against photopic lux **(E)** and melanopic EDI **(F)**. Activity onset (ZT) against photopic lux **(G)** and melanopic EDI **(H)**. Linear regression was used to test for a relationship between the circadian activity parameters and light intensity. The slope of the linear regression line was significantly different from zero in all parameters, and more so against melanopic EDI.

## Results

### Room Lighting and Cage Transmission

The spectral power distribution of the animal facility room light is shown in Figure 1B. The peaks at ∼435, 545, and 610 nm are typical for a modern cool white fluorescent lamp and confirmed wavelength calibration. As expected, the standard cage (Figure 1C) transmits light broadly across the spectrum from 375 to 780 nm, and therefore provides a comparable light environment to the external room (Figure 1D), but at an almost threefold decrease in intensity (measured in μW/cm^2^/s). Conversely, the red cage (Figure 1E) shows a gradual increase in long wavelength transmission, with transmission saturating at 93% from 580 to 780 nm (Figure 1F).

### Activity Across the Digital Ventilate Cage Rack and Between Cages

Figure 2A shows the mean animal locomotion index (ALI) of animals housed in standard (top panel) and red (bottom panel) cages, in the top, middle and bottom rows of the DVC rack. The animal locomotion index is used as a measure of activity, and is plotted in Figure 2A over the course of a week. In both standard and red cages, mice show clear dark phase activity and reduced activity levels during the light phase—in keeping with their nocturnal behavior under laboratory conditions. The most obvious difference is the much higher levels of dark phase activity in mice housed in standard cages compared to red cages. Figures 2B,C are representative actograms of a standard and red cage housed mouse, respectively. Each actogram shows 1 week of activity data, and activity onsets are marked with red triangles (Actogram J). Visual inspection of these actograms suggested that periods of activity and rest may be more consolidated in standard cage versus red cages.

### Circadian Activity Parameters

A range of standard circadian activity parameters are shown in Figure 3, plotted as mean +/− SEM. Two-way repeated-measures ANOVAs were used to test for significant effects of cage type (2 levels—standard and red) and cage position (3 levels—top, middle and bottom) on these key circadian disruption parameters. Light phase activity (%) was higher in the red cages compared to the standard cages across all DVC rack positions, but this effect was not quite statistically significant, *F*_(1.0, 9.0)_ = 4.196, *p* = 0.071 (Figure 3A). This is likely to be due to the high variance observed in light phase activity between animals and cage positions. There was no significant main effect of cage position on light phase activity, *F*_(1.8, 16.4)_ = 0.631, *p* = 0.530, or a significant cage position by cage type interaction, *F*_(2.0, 18.0)_ = 0.026, *p* = 0.975 (Figure 3A). Similarly, standard cage dark phase activity is consistently higher than red cage dark phase activity, but again fails to reach the level of significance, *F*_(1.0, 9.0)_ = 4.196, *p* = 0.071 (Figure 3B). There was no significant main effect of cage position on dark phase activity, *F*_(1.8, 16.4)_ = 0.631, *p* = 0.530, or a significant cage position by cage type interaction, *F*_(2.0, 18.0)_ = 0.026, *p* = 0.975 (Figure 3B). Relative amplitude was higher in standard cages than red cages, across all positions (Figure 3C). However, there was no significant main effect of cage type, *F*_(1.0, 9.0)_ = 4.151, *p* = 0.072, or cage position, *F*_(1.8, 16.3)_ = 0.629, *p* = 0.530. Similarly, there was no significant cage position by cage type interaction, *F*_(2.0, 18.0)_ = 0.023, *p* = 0.975 (Figure 3C).

There was a significant main effect of cage type on activity onset (Figure 3D), with mice housed in red cages starting their activity ∼30 min earlier each day compared to mice in standard cages, *F*_(1.0, 9.0)_ = 8.264, *p* = 0.018. There was no significant main effect of cage position on activity onset, *F*_(2.0, 17.9)_ = 1.623, *p* = 0.225, or significant cage type by cage position interaction, *F*_(2.0, 18.0)_ = 0.074, *p* = 0.929 (Figure 3D); and similar patterns in cage position were seen for both red and standard cage activity onsets.

Finally, the RDI during the light phase was higher in red cage housed mice than in standard cage housed mice, suggesting more irregular activity rhythms, although this was not significant, *F*_(1.0, 9.0)_ = 3.883, *p* = 0.080 (Figure 3E). Similarly, there was no significant main effect of cage position, *F*_(1.9, 17.2)_ = 0.407, *p* = 0.663 or significant cage position by cage type interaction, *F*_(2.0, 18.0)_ = 0.991, *p* = 0.390. Figure 3F shows the RDI calculated for the dark phase, in which there was a significant effect of cage position, *F*_(1.8, 16.6)_ = 5.545, *p* = 0.016, but no significant effect of cage type, *F*_(1.0, 9.0)_ = 0.000, *p* = 0.994, or cage position by cage type interaction, *F*_(2.0, 18.0)_ = 1.41, *p* = 0.270. No significant effects of cage type or position were reported for periodogram power, period, inter-daily stability or intra-daily variability (data not shown).

### Activity Bout Distribution Across the Digital Ventilate Cage Rack and Cage Type

Time-weighted frequency histograms illustrating the distribution of activity bouts in standard and red cages across the DVC rack are shown in Figure 4. Two-way repeated-measures ANOVAs were used to test for significant main effects of cage type (standard and red) and bout length (corresponding to activity bouts of 1, 2, 3, 4–5, 6–10, 11–20, 21–40, > 40 min) on frequency of bouts across top, middle and bottom rack positions. Light phase (top panel) and dark phase (bottom panel) activity data were analyzed separately due to the dramatic differences in activity across the light/dark cycle.

In the light phase, a significant main effect of bout length on frequency of bouts occurred in the top position, *F*_(2.9, 26.0)_ = 122.592, *p* = < 0.0001; middle position, *F*_(2.1, 19.3)_ = 58.549, *p* = < 0.0001, and bottom position, *F*_(1.5, 13.9)_ = 47.561, *p* = < 0.0001. Similarly, in the dark phase, there was a significant main effect of bout length on frequency of bouts across the DVC rack—top position, *F*_(1.6, 15.8)_ = 22.943, *p* = < 0.0001; middle position, *F*_(1.2, 11.0)_ = 13.615, *p* = 0.001; bottom position, *F*_(1.6, 14.1)_ = 17.297, *p* = 0.0003. This is clearly demonstrated in Figure 4, with both standard and red cages across the DVC rack showing higher numbers of shorter activity bouts than longer activity bouts. No significant main effect of cage type on bout frequency was identified in any position, in either of the light or dark phase analyses. However, in the light phase there was a significant cage type by bout length interaction across the top position, *F*_(7.0, 63.0)_ = 9.788, *p* = < 0.0001; middle position, *F*_(7.0, 63.0)_ = 2.331, *p* = 0.035, and bottom position, *F*_(7.0, 63.0)_ = 3.457, *p* = 0.003. This cage type by bout length interaction was only found in the top position of the dark phase analysis, *F*_(7.0, 63.0)_ = 3.080, *p* = 0.007 and not in the middle position, *F*_(7.0, 63.0)_ = 0.658, *p* = 0.707 or the bottom position, *F*_(7.0, 63.0)_ = 0.331, *p* = 0.937. As shown in Figure 4, in all the light phase analyses, and the top position of the dark phase analysis, the frequency of short bouts in standard cages is higher than in the red cages; but as bout length increases, the frequency of bouts becomes higher in red cages than standard cages. This suggests that in the light phase brief bouts of quiet wakefulness predominate in standard cages, whereas longer periods of extended activity occur in red cages. This is consistent with more daytime activity in red cages in comparison with standard cages.

### Relationship Between Circadian Activity Measures and Light Intensity

Light levels vary across a cage rack depending upon its positioning relative to the room lighting. Therefore, light levels for any given cage decrease from the top to the bottom of a cage rack, but also along any row depending upon its proximity to the overhead light (Figure 1A and Supplementary Figure 2). Therefore, light intensity could be more accurately viewed as a continuum across the DVC rack, rather than a categorical variable. Animals housed in the top, middle, or bottom of the cage positions could be exposed to quite different ambient light levels (with a 6.0, 3.5, and 1.6-fold change in top, middle, and bottom positions, respectively, in the standard cages, and a 6.0, 3.4, and 2.2-fold change in top, middle and bottom positions, respectively, in red cages). To account for this variation in home cage light levels, circadian activity measures for every mouse in each position of the DVC rack, were compared against the light intensity for the corresponding cage type and specific position. The relationship between circadian activity measures and light intensity were then explored through a series of linear regression analyses (Figure 5). The circadian disruption measures were correlated with both photopic lux (Figures 5A,C,E,G) and melanopic equivalent daylight illuminance (EDI) (Figures 5B,D,F,H). Not all data points are fully independent—there are 3 data points per mouse (a total of 33 measurements from 11 animals), representing their time in the top, middle, and bottom positions of the DVC rack. However, each plotted value corresponds to a separate week of activity measurements under different lighting conditions.

A significant negative correlation was found between light phase activity (%) and photopic lux, *R*^2^ = 0.247, *p* = 0.003 (Figure 5A), as well as between light phase activity (%) and melanopic irradiance, *R*^2^ = 0.264, *p* = 0.002 (Figure 5B). This is expected since the proportion of total activity occurring during the light phase will be lower in those animals with more strongly entrained activity-wake rhythms (Brown et al., 2019). Interestingly, the intensity of light during the light phase not only influences light phase activity, but also the level of dark phase activity. As shown in Figure 5C a significant positive correlation was found between dark phase activity (%) and photopic lux, *R*^2^ = 0.247, *p* = 0.003, as well as with melanopic irradiance, *R*^2^ = 0.264, *p* = 0.002 (Figure 5D). Relative amplitude is related to both light and dark phase activity. A significant positive correlation was found between relative amplitude and photopic lux, *R*^2^ = 0.243, *p* = 0.004 (Figure 5E) and melanopic irradiance, *R*^2^ = 0.260, *p* = 0.002 (Figure 5F). The correlations between light intensity and timing of activity onset are more significant than the other three measures, with a significant positive correlation of *R*^2^ = 0.276, *p* = 0.002 found between timing of activity onset and photopic lux (Figure 5G), and melanopic irradiance, *R*^2^ = 0.322, *p* = 0.001 (Figure 5H).

For all parameters, the correlations between melanopic irradiance and circadian activity measures are stronger than with photopic lux. This is expected given that photopic lux is based on cone-mediated vision (weighted to 555 nm) in a standard human observer (Al Enezi et al., 2011) and therefore is not relevant to the mouse visual system, which only possesses an M-cone with a peak sensitivity at 508 nm (Sun et al., 1997). Melanopic irradiance, with a peak sensitivity at ∼480 nm, has been widely proposed as a unit of circadian light intensity (Al Enezi et al., 2011; Lucas et al., 2014) and is likely to be more strongly correlated to measures of entrainment due to the role of melanopsin ipRGCs in circadian entrainment.

## Discussion

Here we show that light intensity differs markedly across a standard IVC rack, with over a 15-fold difference between highest and lowest intensities in standard cages and over a 10-fold lower intensity in red cages. This variation results from the relative position of light sources to the IVC rack as well as other features of the room layout. The light environment experienced by laboratory animals will therefore be influenced not only by the animal facility lighting, but by the specific position of the cage within any IVC rack. Similar phenomena have been reported before, such as in Weihe et al. (1969) where an 83-fold difference in light intensity was shown between transparent cages at the top and bottom of a rack. Furthermore, cage composition can impact the spectrum of light reaching an animal; an effect which has been explored previously in the context of nude rats housed in transparent, blue, and amber cages (Dauchy et al., 2013). Disrupted rhythms in measures of endocrine metabolism and physiology, such as plasma corticosterone levels, were reported for animals housed in blue and amber cages compared to transparent cages (Dauchy et al., 2013).

There is an extensive literature showing the potent effects of light on physiology and behavior. Therefore, we predicted that the differences in light environment across cage type and position in our study would impact key circadian activity measures. Indeed, mice housed in red cages and therefore under a lower light intensity at each position, started their activity significantly earlier (∼30 min) than mice housed in standard cages. Similarly, red-caged mice showed a higher level of light phase activity, lower level of dark phase activity as well as a lower relative amplitude than the control group. Whilst these latter differences were not significant, they are all features of less robust circadian entrainment (Brown et al., 2019). Furthermore, a significant interaction of cage type and bout length was seen in the light phase activity bout analysis, with red cages showing longer periods of extended activity, consistent with greater light phase activity in red cages than standard cages.

Our data also demonstrate that simply categorizing light intensity by row height (top, middle, and bottom) in the IVC rack may be overly simplistic, as substantial variation in light intensity within each row was also observed (Table 1 and Supplementary Figure 2). Light intensity should be viewed as a continuum, and in this way, both standard and red cage data can be analyzed together. Linear regression was used to test for a significant relationship between both photopic and melanopic irradiance and the key circadian parameters (Figure 5). The strength of circadian entrainment, as described by key parameters, was found to increase with increasing light intensity. The slope of the linear regression line was significantly different from zero in all parameters, reflecting that light intensity explained a greater proportion of the variation in the data than a horizontal line through the mean. Although the *R*^2^ values are significant, they are quite low; indicating that light intensity does not explain all the variance seen in the key circadian parameters. The *R*^2^-values were higher for melanopic irradiance than photopic lux across all circadian parameters, which is consistent with the key role of melanopsin ipRGCs in circadian entrainment (Al Enezi et al., 2011).

Although mice housed in red cages showed earlier activity onsets, all mice in the IVC rack were still able to successfully entrain to the light dark cycle. This is not surprising given that previous work has shown that mice should be able to entrain down to light levels as low as 0.01 photopic lux (Ebihara and Tsuji, 1980; Foster and Helfrich-Förster, 2001; Butler and Silver, 2011), whereas the lowest light intensity recorded in our IVC rack was only 1.8 photopic lux. As such, the light levels across an IVC rack should be well above the threshold for circadian entrainment. Furthermore, our data shows that red cages are not equivalent to darkness for mice. Whilst mice lack a long-wave sensitive (red) cone, they are still able to detect and entrain to red light if the intensity is sufficiently high (Peirson et al., 2018).

The circadian parameters shown in Figure 5 were also analyzed against α-opic irradiance values for the additional mouse retinal photoreceptors, including the UV-sensitive short-wavelength sensitive cones (S-cones), medium-wave sensitive cones (M-cones), and rods. As expected, based upon the known role of melanopsin in circadian entrainment, melanopic irradiance correlated most strongly with light phase activity, dark phase activity and relative amplitude (Supplementary Table 2). Photopic lux correlated least strongly, which adds support to the argument that this measure is not as relevant as melanopic irradiance when studying mice, since it is based upon human visual sensitivity (Al Enezi et al., 2011; Brown et al., 2013). Interestingly, activity onset showed a slightly higher correlation with S-cone-opic irradiance values than melanopic irradiance (Supplementary Table 2). As mice were housed under a light/dark cycle with ramped light transitions, different photoreceptors may play a role in the timing of activity onset at the light to dark transition. Under these conditions, the dynamically changing light intensity may be a sensory task that favors cones. If this is indeed the case, it is intriguing that S-cone-opic irradiance correlates more strongly than M-cone-opic irradiance. Whilst previous work has shown that S-cones play a role in circadian entrainment (van Oosterhout et al., 2012), M-cones can also contribute (Lall et al., 2010). In the current study, the greater correlation with S-cone-opic irradiance may reflect the dorsal-ventral gradient in cone opsin, with higher S-cone expression in the ventral retina, which will receive more light from the upper visual field (Hughes et al., 2013). However, these findings are preliminary and further research into the role of specific photoreceptors in different aspects of home cage activity is needed.

Our study has several important limitations. It was originally designed to be completely counterbalanced, however, the loss of one male mouse due to fighting injuries before the start of the experiment resulted in an unbalanced design. In addition, because of row and column effects on cage light levels and the use of males and females, greater variance was observed in circadian parameters than under comparable studies in light-controlled chambers (Albrecht and Russell, 2002; Jud et al., 2005; Hughes et al., 2015). As a result, greater statistical power would be beneficial to detect more subtle changes. As is routinely used in circadian studies, animals were singly housed to allow activity monitoring to be attributed to a single animal. As the cage is the experimental unit in mouse studies, this reduced the number of animals used, but could potentially affect home cage behavior and thermoregulation (Festing et al., 2016).

Light exerts potent effects on the physiology and behavior of mice, including activity levels (Albrecht and Russell, 2002; Jud et al., 2005; Hughes et al., 2015), sleep and arousal (Pilorz et al., 2016), body temperature (McGuire et al., 1973), melatonin production (Brainard et al., 1984), and corticosterone secretion (Ishida et al., 2005). Therefore, differences in home cage light levels across experimental cages are a source of potential biological variability. This may be particularly relevant for tests of exploratory activity, anxiety, and photophobia; but also has the potential to affect cognitive performance (Tam et al., 2016) since prior sleep has been suggested to drive variation in this (Tam et al., 2021). If this is indeed the case, it may pose a potential problem for reproducibility in animal research, especially since different animal facilities, and even separate rooms within the same facility, will differ in the relative positioning of cage racks and room lighting. Furthermore, the use of different light sources and cage types may also affect the spectral composition of the light source. A good example of this is provided by comparison of fluorescence fixtures to white LEDs, with white LEDs providing relatively less short-wavelength light and thus an S-cone depleted light environment. Furthermore, the age of cages (with repeated cycles of washing) and the age of lighting fixtures may also affect the light conditions experienced by animals. Together these aspects of facility design mean that light levels experienced by laboratory mice may vary dramatically between studies.

To try and reduce this potential source of variation, researchers should factor cage position into experimental design to account for differences in light intensity experienced between animals. Ideally, researchers should also report the light levels experienced by animals within the cage, rather than just the room lighting. More detailed characterization of the lighting is helpful, particularly spectral power distributions, as these enable the effects of light on the different photoreceptors of the mouse retina to be determined (Lucas et al., 2014). However, in the absence of a spectrophotometer, reporting the type of lighting (and manufacturer), and photopic lux can be helpful. Lux meters are cheap and widely available and can provide a simple measure of light intensity. Whilst lux is based on human perceived brightness and is not directly relevant to the visual system of mice, it provides an approximation of intensity. Furthermore, for any given light source, the ratio of melanopic to photopic lux (M/P ratio) can be determined and used to convert photopic lux measurements to melanopic irradiance. For example, the MP ratio of daylight is 1.0, but for a cool white fluorescent light source this may be 0.56. As such, 100 photopic lux from such a light will give 56 melanopic irradiance. In the future, technological developments may help standardization in this area, for example, cage racks or cages with inbuilt lighting.

## Conclusion

In conclusion, this study highlights how light can vary dramatically across a single IVC rack, and the subsequent effects this can have on a range of circadian activity measures such as activity levels and the timing of activity onset. Given the widespread effects of light on visual and circadian physiology and behavior, such differences may reflect a source of uncharacterized variability in mouse studies and may be important for improving reproducibility.

## Data Availability Statement

The raw data supporting the conclusions of this article will be made available by the authors, without undue reservation.

## Ethics Statement

The animal study was reviewed and approved by the Clinical Medicine Animal Welfare and Ethical Review Body (AWERB), University of Oxford.

## Author Contributions

LCES and SNP designed the study with help from SKET. ST set up the DVC system and provided technical assistance. LCES conducted all experiments with help from ST and SKET. LCES analyzed all data with input from ST, SKET, and SNP. LCES and SNP prepared the manuscript with input from all authors. All authors approved the manuscript.

## Conflict of Interest

The authors declare that the research was conducted in the absence of any commercial or financial relationships that could be construed as a potential conflict of interest.

## Publisher’s Note

All claims expressed in this article are solely those of the authors and do not necessarily represent those of their affiliated organizations, or those of the publisher, the editors and the reviewers. Any product that may be evaluated in this article, or claim that may be made by its manufacturer, is not guaranteed or endorsed by the publisher.
