# Supplementary material for: Effects of Cage Position and Light Transmission on Home Cage Activity and Circadian Entrainment in Mice
Source: Front Neurosci. 2022 Jan 10;15:832535. doi: 10.3389/fnins.2021.832535 (PMC8784806; doi:10.3389/fnins.2021.832535)
Supplement: Supplementary file 1 [file Presentation_1.PPTX]

## Slide 1
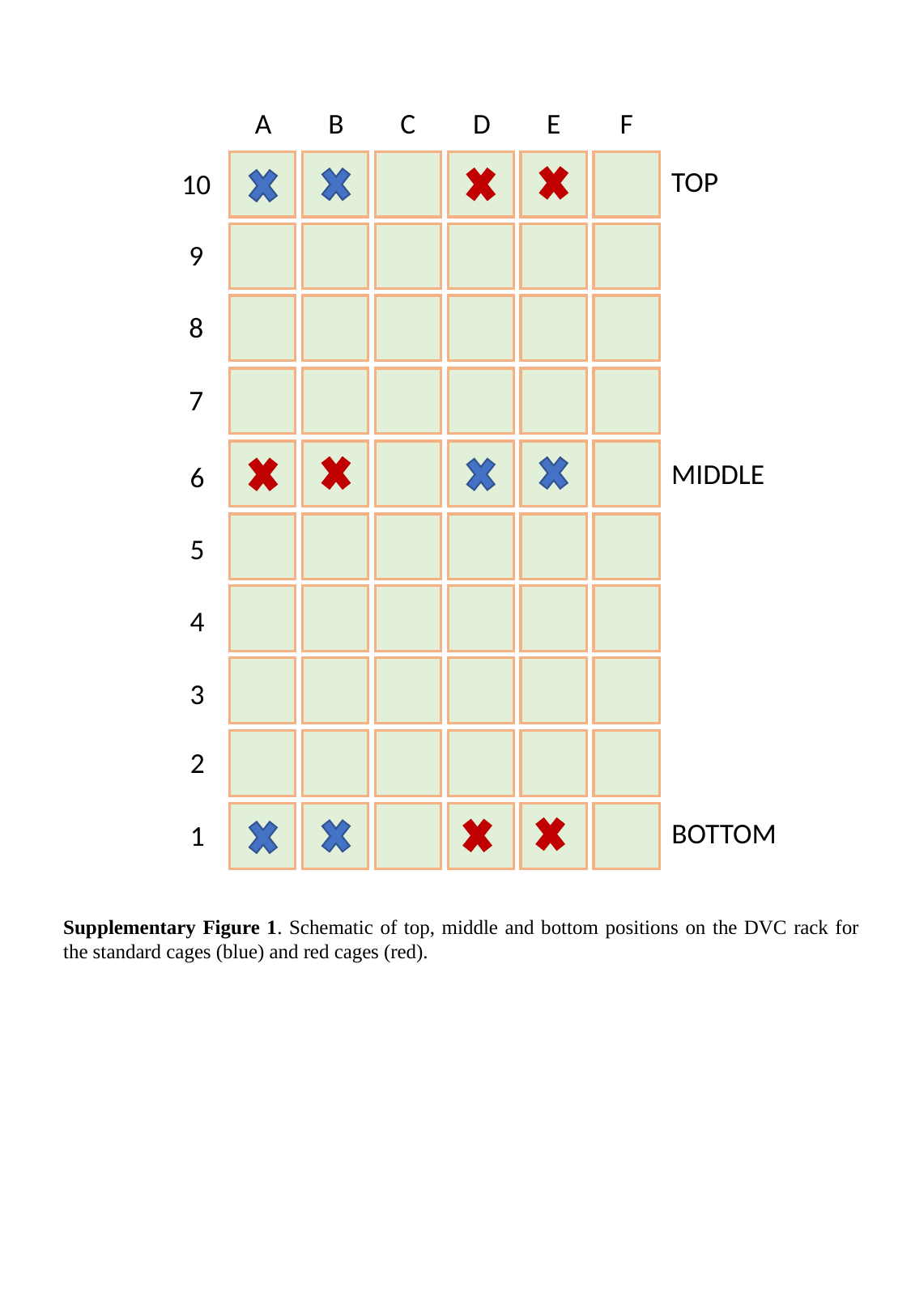

A
B
C
D
E
F
10
9
8
7
6
5
4
3
2
1
TOP
MIDDLE
BOTTOM
Supplementary Figure 1. Schematic of top, middle and bottom positions on the DVC rack for the standard cages (blue) and red cages (red).

## Slide 2
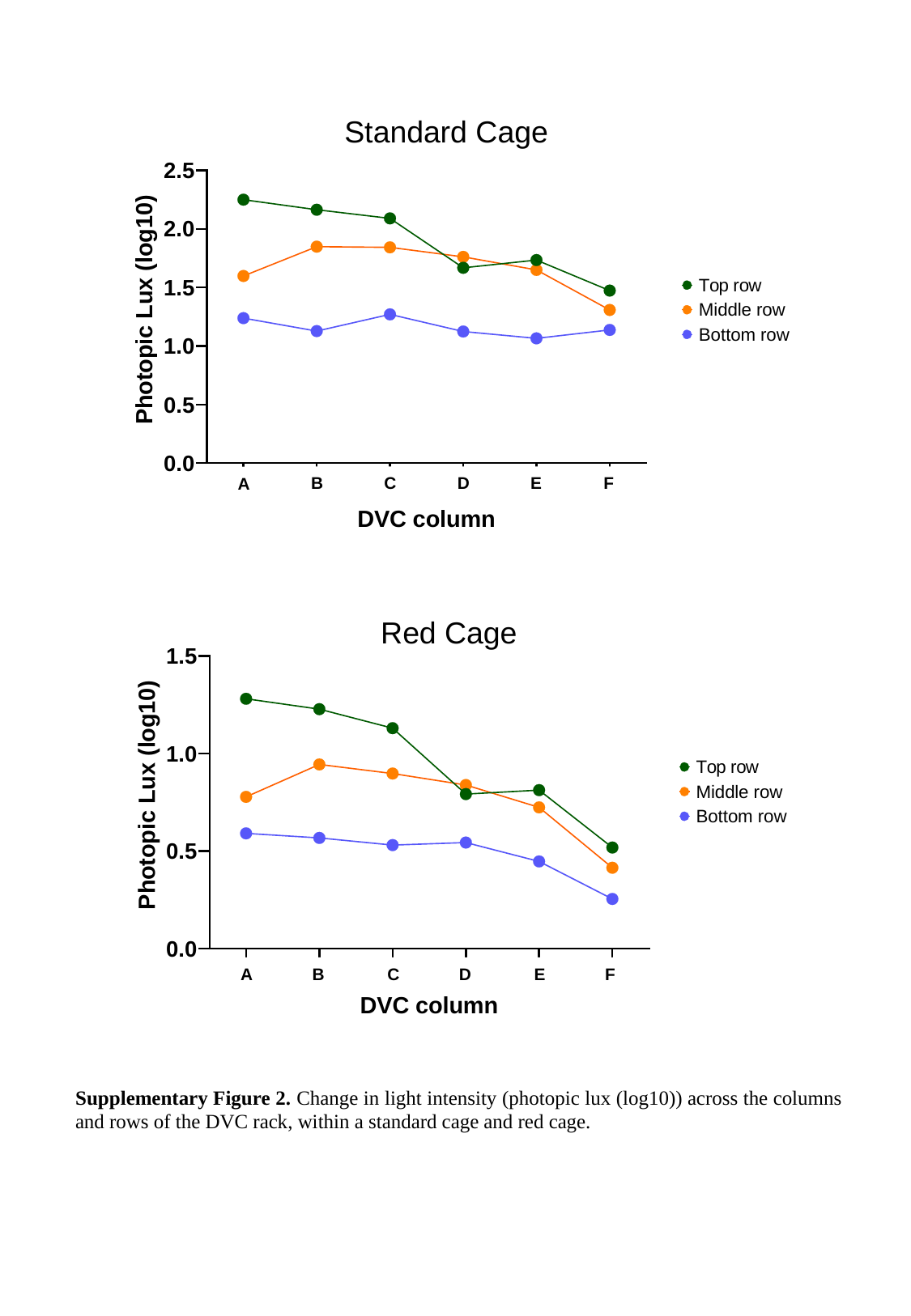

Standard Cage
F
D
E
B
C
A
Red Cage
C
D
E
F
A
B
Supplementary Figure 2. Change in light intensity (photopic lux (log10)) across the columns and rows of the DVC rack, within a standard cage and red cage.

## Slide 3
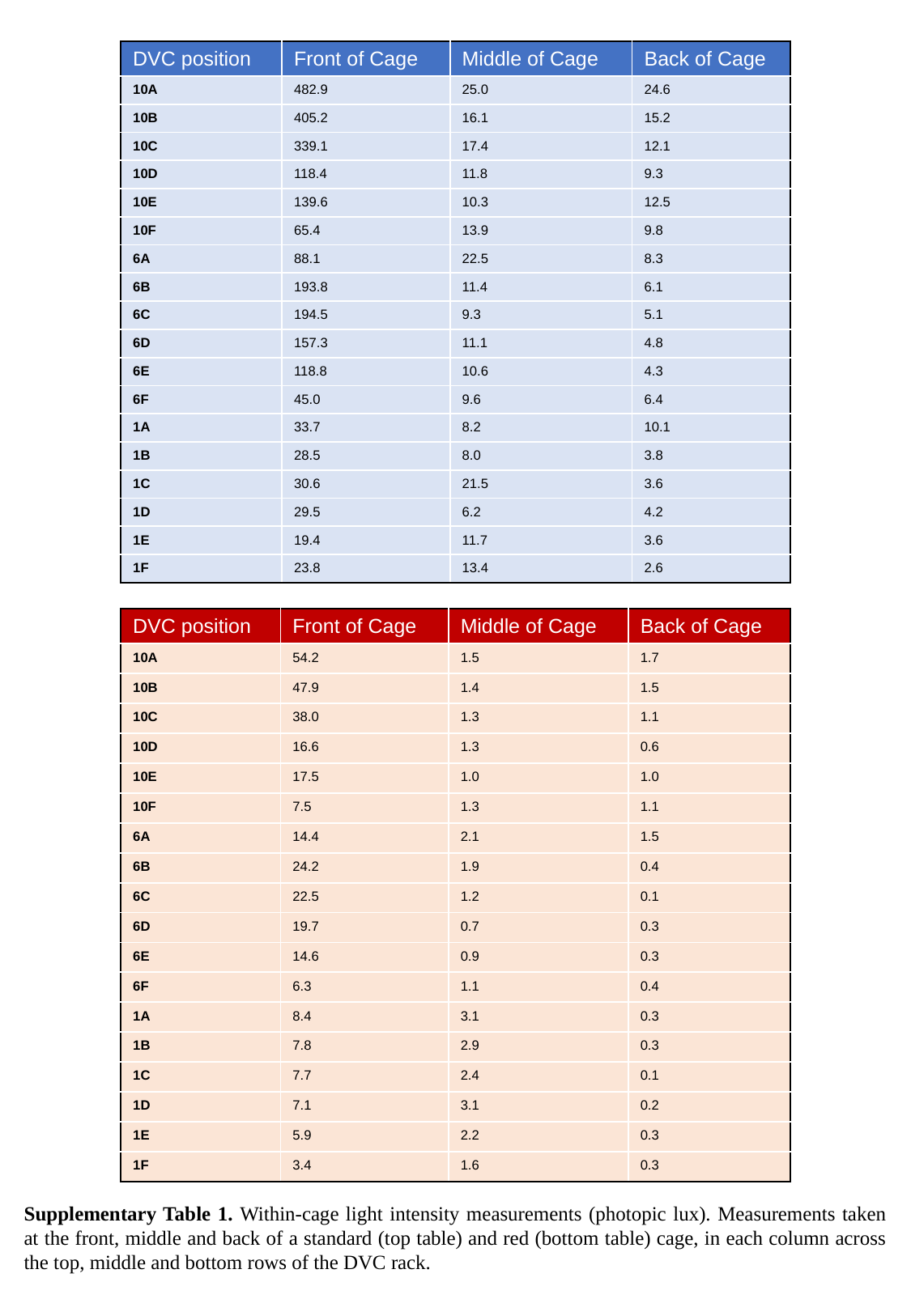

| DVC position | Front of Cage | Middle of Cage | Back of Cage |
| --- | --- | --- | --- |
| 10A | 482.9 | 25.0 | 24.6 |
| 10B | 405.2 | 16.1 | 15.2 |
| 10C | 339.1 | 17.4 | 12.1 |
| 10D | 118.4 | 11.8 | 9.3 |
| 10E | 139.6 | 10.3 | 12.5 |
| 10F | 65.4 | 13.9 | 9.8 |
| 6A | 88.1 | 22.5 | 8.3 |
| 6B | 193.8 | 11.4 | 6.1 |
| 6C | 194.5 | 9.3 | 5.1 |
| 6D | 157.3 | 11.1 | 4.8 |
| 6E | 118.8 | 10.6 | 4.3 |
| 6F | 45.0 | 9.6 | 6.4 |
| 1A | 33.7 | 8.2 | 10.1 |
| 1B | 28.5 | 8.0 | 3.8 |
| 1C | 30.6 | 21.5 | 3.6 |
| 1D | 29.5 | 6.2 | 4.2 |
| 1E | 19.4 | 11.7 | 3.6 |
| 1F | 23.8 | 13.4 | 2.6 |
| DVC position | Front of Cage | Middle of Cage | Back of Cage |
| --- | --- | --- | --- |
| 10A | 54.2 | 1.5 | 1.7 |
| 10B | 47.9 | 1.4 | 1.5 |
| 10C | 38.0 | 1.3 | 1.1 |
| 10D | 16.6 | 1.3 | 0.6 |
| 10E | 17.5 | 1.0 | 1.0 |
| 10F | 7.5 | 1.3 | 1.1 |
| 6A | 14.4 | 2.1 | 1.5 |
| 6B | 24.2 | 1.9 | 0.4 |
| 6C | 22.5 | 1.2 | 0.1 |
| 6D | 19.7 | 0.7 | 0.3 |
| 6E | 14.6 | 0.9 | 0.3 |
| 6F | 6.3 | 1.1 | 0.4 |
| 1A | 8.4 | 3.1 | 0.3 |
| 1B | 7.8 | 2.9 | 0.3 |
| 1C | 7.7 | 2.4 | 0.1 |
| 1D | 7.1 | 3.1 | 0.2 |
| 1E | 5.9 | 2.2 | 0.3 |
| 1F | 3.4 | 1.6 | 0.3 |
Supplementary Table 1. Within-cage light intensity measurements (photopic lux). Measurements taken at the front, middle and back of a standard (top table) and red (bottom table) cage, in each column across the top, middle and bottom rows of the DVC rack.

## Slide 4
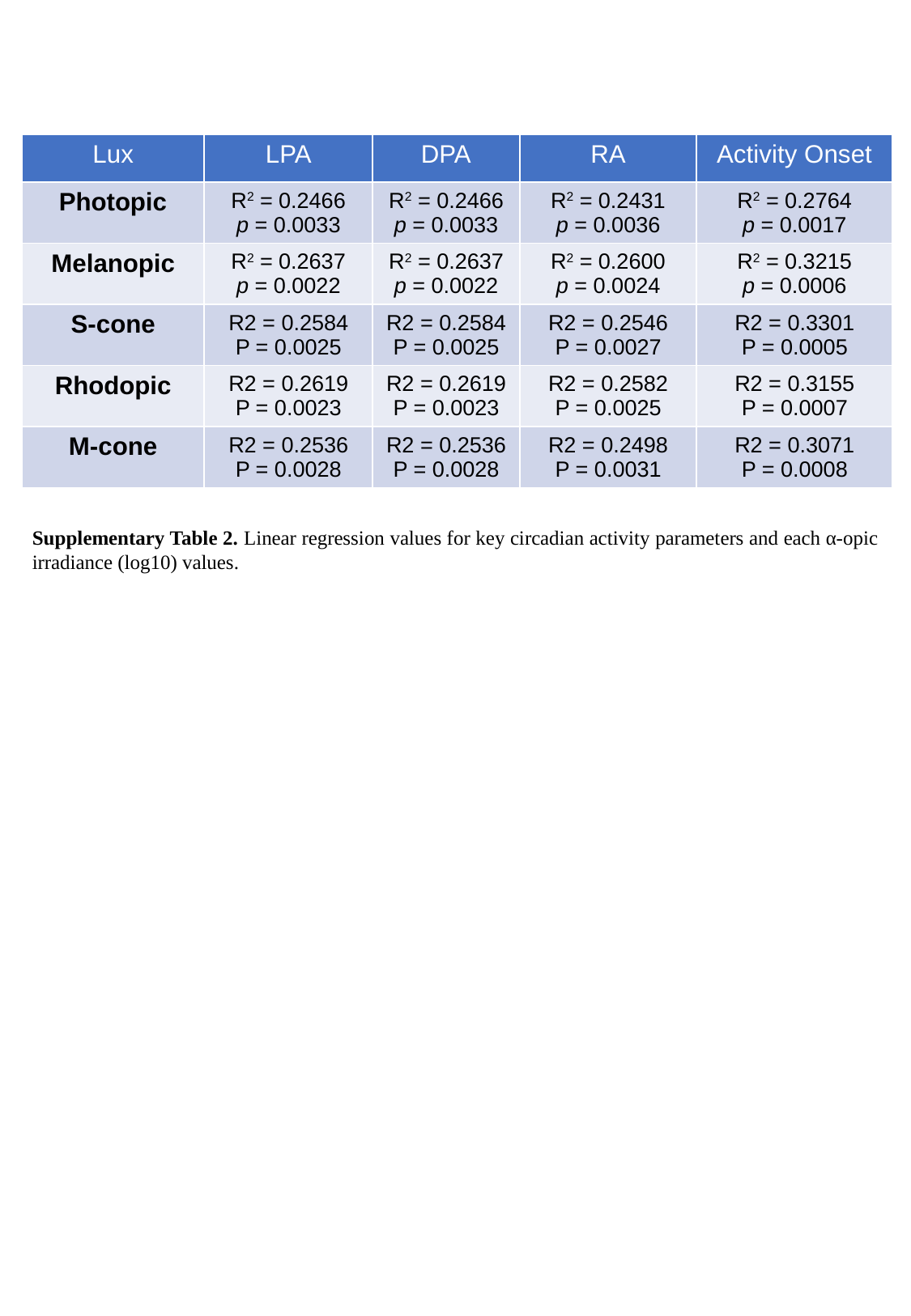

| Lux | LPA | DPA | RA | Activity Onset |
| --- | --- | --- | --- | --- |
| Photopic | R2 = 0.2466 p = 0.0033 | R2 = 0.2466 p = 0.0033 | R2 = 0.2431 p = 0.0036 | R2 = 0.2764 p = 0.0017 |
| Melanopic | R2 = 0.2637 p = 0.0022 | R2 = 0.2637 p = 0.0022 | R2 = 0.2600 p = 0.0024 | R2 = 0.3215 p = 0.0006 |
| S-cone | R2 = 0.2584 P = 0.0025 | R2 = 0.2584 P = 0.0025 | R2 = 0.2546 P = 0.0027 | R2 = 0.3301 P = 0.0005 |
| Rhodopic | R2 = 0.2619 P = 0.0023 | R2 = 0.2619 P = 0.0023 | R2 = 0.2582 P = 0.0025 | R2 = 0.3155 P = 0.0007 |
| M-cone | R2 = 0.2536 P = 0.0028 | R2 = 0.2536 P = 0.0028 | R2 = 0.2498 P = 0.0031 | R2 = 0.3071 P = 0.0008 |
Supplementary Table 2. Linear regression values for key circadian activity parameters and each α-opic irradiance (log10) values.
